# Circulating Organic Acid Profiles in Non-Ischaemic Cardiomyopathy: A Case–Control Study

**DOI:** 10.3390/jcdd13080350

**Published:** 2026-07-27

**Authors:** Yasemin Behram Kandemir, Ismail Koyuncu, Unal Guntekin, Veysel Tosun, Necmettin Korucuk, Eyyup Tusun, Ersin Doganozu

**Affiliations:** 1 Department of Anatomy, Faculty of Medicine, Istanbul Aydin University, Istanbul 34295, Turkey; ybehramkandemir@gmail.com; 2 Department of Medical Biochemistry, Faculty of Medicine, Harran University, Sanliurfa 63050, Turkey; ikoyuncu@harran.edu.tr; 3 Department of Cardiology, Akdeniz University, Antalya 07070, Turkey; unalguntekin@akdeniz.edu.tr; 4 Department of Cardiology, Mehmet Akif Inan Education and Research Hospital, Sanliurfa 63050, Turkey; veyseltosun8810@gmail.com (V.T.); eyubtusun@hotmail.com (E.T.); 5 Department of Cardiology, Antalya City Hospital, Antalya 07080, Turkey; necmettinmd@gmail.com

**Keywords:** metabolomics, cardiomyopathy, organic acids, metabolic remodeling

## Abstract

Metabolic remodeling is increasingly recognized as a key component of cardiomyopathy, yet the circulating organic acid profile associated with this condition remains incompletely characterized. In this case–control study, we investigated differences in circulating organic acid profiles between patients with cardiomyopathy and control participants using targeted liquid chromatography–tandem mass spectrometry (LC-MS/MS). A total of 160 participants were enrolled, including 80 patients with cardiomyopathy and 80 age- and sex-comparable control participants. Circulating organic acids were quantitatively analyzed, and between-group differences were assessed using the two-sided Mann–Whitney *U* test with Benjamini–Hochberg false discovery rate correction; effect sizes were estimated using Cliff’s delta. Detection rates were also examined because several metabolites included values below the assay limit of detection. Sixteen metabolites remained significant after correction. Among these, 3-hydroxyisovaleric acid demonstrated the largest increase, followed by 2-oxoglutaric acid and 2-methylcitric acid. Additional elevations were observed in citric acid, malic acid, N-acetylaspartic acid, fumaric acid, and suberic acid, whereas 2-oxoadipic acid was reduced. These alterations are consistent with perturbations in mitochondrial intermediary metabolism, branched-chain amino acid catabolism, ketone body metabolism, and tricarboxylic acid cycle-related pathways. However, because comorbidities, renal function, glycemic status, and medication use differed between groups, the findings should be interpreted as phenotype-associated metabolic signals rather than cardiomyopathy-specific causal effects or validated diagnostic biomarkers. In conclusion, targeted LC-MS/MS-based organic acid profiling reveals an altered circulating metabolic pattern in cardiomyopathy, although further prospective studies with individual-level adjustment and external validation are required to determine its clinical relevance.

## 1. Introduction

Cardiomyopathies comprise a heterogeneous group of myocardial disorders characterized by structural and/or functional abnormalities of the myocardium that cannot be fully explained by coronary artery disease, hypertension, valvular disease, or congenital heart disease alone [1]. Despite major advances in imaging, molecular diagnostics, and heart failure management, cardiomyopathies remain associated with substantial morbidity and mortality due to progressive ventricular dysfunction, arrhythmias, and heart failure-related complications [1]. In clinical practice, conventional phenotyping largely relies on echocardiographic findings, functional status, and, when available, genetic characterization. However, these approaches do not fully capture the dynamic biochemical changes accompanying myocardial injury and disease progression. Accordingly, there is increasing interest in identifying circulating molecular signatures that may complement conventional clinical assessment and provide additional insight into disease biology [2].

Among the emerging mechanisms implicated in cardiomyopathy, metabolic remodeling has gained particular attention as a key component of myocardial dysfunction [3,4]. The healthy heart is metabolically flexible and can adapt substrate utilization according to energetic demand and substrate availability. In contrast, diseased myocardium shows impaired substrate flexibility, altered mitochondrial oxidative metabolism, and disturbances in intermediary metabolic pathways, all of which may contribute to energetic inefficiency and contractile dysfunction [3,4]. Experimental and clinical evidence increasingly supports the view that such metabolic shifts are not merely secondary epiphenomena, but rather integral features of myocardial remodeling and heart failure pathophysiology [3,4]. In this context, the characterization of circulating metabolites may offer a biologically meaningful window into systemic and myocardial metabolic perturbations.

Metabolomics has emerged as a powerful systems-level approach for capturing biochemical alterations associated with cardiovascular disease [2]. In particular, cardiovascular metabolomics enables the identification of circulating metabolite patterns that may reflect altered substrate utilization, mitochondrial stress, redox imbalance, and maladaptive energy metabolism [2,5]. Previous studies have demonstrated that heart failure and related cardiometabolic conditions are associated with distinct metabolic fingerprints, supporting the concept that circulating metabolites may improve disease phenotyping and mechanistic interpretation [5,6]. These observations are especially relevant in cardiomyopathy, where substantial clinical heterogeneity suggests that conventional structural classification alone may not fully represent underlying pathobiology.

Within the metabolome, organic acids occupy central positions in intermediary metabolism, linking glycolysis, branched-chain amino acid catabolism, anaplerotic flux, and the tricarboxylic acid cycle. Because of this central metabolic role, changes in circulating organic acid levels may reflect impaired oxidative substrate handling, mitochondrial dysfunction, and altered metabolic flexibility [4,5]. Several metabolites within this biochemical class are closely related to mitochondrial pathways that have been implicated in myocardial dysfunction and heart failure phenotypes [4,5,6]. From an analytical perspective, targeted metabolomics provides a robust framework for the quantitative assessment of predefined metabolites with clear biochemical annotation, and liquid chromatography–tandem mass spectrometry (LC–MS/MS) is particularly well suited for this purpose because it allows sensitive and selective measurement of low-abundance intermediary metabolites, including multiple circulating organic acids [7,8].

Recent metabolomics studies in heart failure have highlighted the potential clinical relevance of circulating metabolite profiling for phenotypic subclassification, prognostic stratification, and mechanistic exploration [9,10,11,12,13,14]. However, most prior targeted studies have evaluated broad or composite metabolite panels that include amino acids, acylcarnitines, lipids, and ketone-related metabolites, while only a limited number of investigations have specifically focused on a dedicated circulating organic acid profile [9,10,13,14]. As a result, the contribution of organic acid perturbations to the metabolic phenotype of cardiomyopathy remains insufficiently characterized. This gap is clinically important because focused analysis of organic acids may reveal pathway-level disturbances that are less apparent in broader but less mechanistically centered metabolomic panels.

Against this background, we designed the present case–control study to characterize the circulating organic acid profile associated with cardiomyopathy using targeted LC–MS/MS. We hypothesized that patients with cardiomyopathy would exhibit a distinct circulating organic acid pattern compared with control participants without known cardiomyopathy or structural heart disease. However, these controls were not free of all cardiometabolic comorbidities, and this was considered in the interpretation of the findings. Therefore, in a cohort comprising 80 patients with cardiomyopathy and 80 control participants, we aimed to define the circulating organic acid landscape associated with cardiomyopathy and to identify the metabolites most strongly associated with the cardiomyopathy phenotype.

## 2. Materials and Methods

### 2.1. Study Design and Population

This case–control study included a total of 160 participants, comprising 80 patients with cardiomyopathy and 80 control participants. Patients with cardiomyopathy were recruited from the Departments of Cardiology at Akdeniz University Hospital and Mehmet Akif İnan Training and Research Hospital between October 2022 and April 2024. The diagnosis of cardiomyopathy was established by experienced cardiologists based on clinical evaluation, transthoracic echocardiography, and standard diagnostic criteria. Control participants were recruited from individuals without known cardiomyopathy or structural heart disease; however, they were not necessarily free of all cardiometabolic comorbidities.

Participants with acute infectious or inflammatory conditions, active malignancy, severe hepatic failure, end-stage renal disease, or incomplete clinical/biochemical data were excluded. Demographic and clinical characteristics, including age, sex, anthropometric measurements, comorbidities, medication use, and routine laboratory findings, were recorded at study entry.

Cardiomyopathy was defined according to contemporary clinical guidance as a structural and/or functional myocardial abnormality not explained by significant coronary artery disease or abnormal loading conditions sufficient to account for the observed myocardial dysfunction. Ischemic cardiomyopathy was excluded based on clinical history and, when clinically indicated, coronary angiography or non-invasive coronary imaging. Accordingly, the study cohort predominantly represented non-ischemic cardiomyopathy (NICM). The presence of coronary artery disease did not necessarily indicate ischemic cardiomyopathy; patients were classified as NICM when myocardial dysfunction could not be explained by coronary artery disease distribution or prior myocardial infarction. Most patients exhibited a dilated cardiomyopathy phenotype within the NICM spectrum. Non-ischemic cardiomyopathy was defined as myocardial dysfunction not attributable to obstructive coronary artery disease and not developing secondary to prior myocardial infarction. This study was reported in accordance with the STROBE guidelines. Patients and/or the public were not involved in the design, conduct, reporting, or dissemination plans of this research.

### 2.2. Clinical Characteristics and Laboratory Variables

Baseline demographic, clinical, and routine laboratory data were recorded for all participants at study entry. These variables included age, sex, body mass index, comorbidities, medication use, and standard biochemical parameters obtained from hospital records and/or routine blood testing. Continuous variables were tested for normality using the Shapiro–Wilk test and are presented as mean ± standard deviation or median (interquartile range), as appropriate. Categorical variables are expressed as a number (percentage). Between-group comparisons for clinical and laboratory variables were performed using the independent-samples *t* test or Mann–Whitney *U* test for continuous variables, and the chi-square test or Fisher’s exact test for categorical variables, as appropriate.

### 2.3. Ethical Approval

The study protocol was approved by the Harran University Clinical Research Ethics Committee (decision no. 22/01/03, 10 January 2022). All procedures were conducted in accordance with the principles of the Declaration of Helsinki. Written informed consent was obtained from all participants prior to inclusion in the study.

### 2.4. Blood Sampling and Pre-Analytical Processing

Peripheral venous blood samples were collected under standardized clinical laboratory conditions from all participants. After collection into plasma tubes, samples were centrifuged according to the routine laboratory protocol, and the obtained plasma was aliquoted and stored at −80 °C until analysis. Repeated freeze–thaw cycles were avoided. Exact fasting duration, time of day, storage duration, and analytical batch randomization data were not uniformly available for all participants; this limitation was considered when interpreting organic acids and ketone-related metabolites.

### 2.5. Targeted Organic Acid Profiling by LC–MS/MS

Circulating organic acids were quantified by targeted liquid chromatography-tandem mass spectrometry (LC-MS/MS) using a commercially available assay kit (Jasem, Sem Laboratuvar Cihazları Paz. San. ve Tic. A.Ş., Istanbul, Turkey). Sample preparation was performed in accordance with the manufacturer’s instructions and the validated laboratory protocol used at our center, including deproteinization/extraction and, where applicable, the addition of internal standards. Chromatographic separation was performed using the commercial LC-MS/MS kit, and detection was carried out on a Shimadzu LCMS-8045 triple-quadrupole mass spectrometer (Shimadzu Corporation, Kyoto, Japan) coupled to a Shimadzu HPLC system, operating in multiple reaction monitoring (MRM) mode. Metabolites were identified based on retention times and characteristic ion transitions and were quantified using calibration-based analysis. Analytical quality assurance included calibration standards, internal quality controls, and blank samples within each analytical run. Detailed information on the analytical platform, including instrument settings, column characteristics, mobile phases, and analyte panel composition, is provided in Supplementary Table S1. Values reported as 0.000 indicate concentrations below the assay limit of detection (LOD), rather than rounded non-zero concentrations. These values were retained as reported for the primary non-parametric analyses, and detection rates were summarized to clarify when findings reflected differences in detectability as well as concentration distribution.

### 2.6. Statistical Analysis

Statistical analyses were performed in Python version 3.11.9. Continuous variables were assessed for normality by visual inspection and the Shapiro–Wilk test. As metabolite data were predominantly non-normally distributed and included values below the LOD, between-group comparisons for circulating organic acids were performed using the two-sided Mann–Whitney *U* test. Categorical variables were compared using the chi-square test or Fisher’s exact test, as appropriate. To account for multiple comparisons across the metabolite panel, raw *p*-values were adjusted using the Benjamini–Hochberg false discovery rate (FDR) procedure. Metabolites with an FDR-adjusted q value < 0.05 were considered statistically significant. In addition to significance testing, effect sizes were estimated using Cliff’s delta to quantify the magnitude and direction of between-group differences. For metabolites with frequent below-LOD values, the percentage of detectable measurements (>0) was calculated in each group and detection frequencies were compared using Fisher’s exact test with FDR correction. Because the available analysis dataset did not contain complete individual-level linkage between metabolomic measurements and all clinical covariates, the primary metabolite comparisons were not adjusted for comorbidities, renal function, glycemic status, or medication use. Therefore, all metabolite differences are interpreted as unadjusted phenotype-associated findings. Exploratory ROC analyses were performed for the four selected metabolites included in the exploratory ROC panel and for a combined 4-metabolite panel. Single-metabolite AUCs were calculated as univariable exploratory estimates, whereas the combined panel was generated using 5-fold cross-validated logistic regression using only the four selected metabolites. Clinical variables and medication use were not included in the ROC models. For metabolites with a high proportion of below-LOD values, Fisher’s exact tests were used to compare detection frequencies between groups. These analyses were used to support the interpretation of Mann–Whitney results, particularly when median concentrations were 0.000 in one or both groups. As a targeted sensitivity analysis, the four metabolites included in the exploratory ROC panel—3-hydroxyisovaleric acid, 2-oxoglutaric acid, 2-methylcitric acid, and 2-oxoadipic acid—were further summarized using rank-based between-group comparisons, Cliff’s delta effect sizes, detection-rate analyses, and exploratory ROC metrics.

## 3. Results

### 3.1. Study Population and Baseline Characteristics

A total of 160 participants were included in the study, comprising 80 patients with cardiomyopathy and 80 control participants without known cardiomyopathy or structural heart disease. Compared with control participants, patients with cardiomyopathy had a higher burden of cardiometabolic comorbidities and medication use, as shown in Table 1. Age, sex distribution, body mass index, smoking status, and blood pressure values were broadly comparable between groups, whereas heart rate was higher in the cardiomyopathy group. Patients with cardiomyopathy also showed a higher prevalence of cardiovascular comorbidities, including hypertension, diabetes mellitus, dyslipidemia, coronary artery disease, atrial fibrillation, and chronic kidney disease. In routine laboratory testing, the cardiomyopathy group had lower hemoglobin and HDL-cholesterol levels, alongside higher white blood cell count, creatinine, fasting glucose, triglycerides, and lower estimated glomerular filtration rate. As expected, heart failure-related medical therapies, including ACE inhibitor/ARB, ARNI, beta-blocker, mineralocorticoid receptor antagonist, SGLT2 inhibitor, and loop diuretic use, were more frequent in the cardiomyopathy group (Table 1).

### 3.2. Differential Circulating Organic Acid Profile in Cardiomyopathy

**Figure 1:**
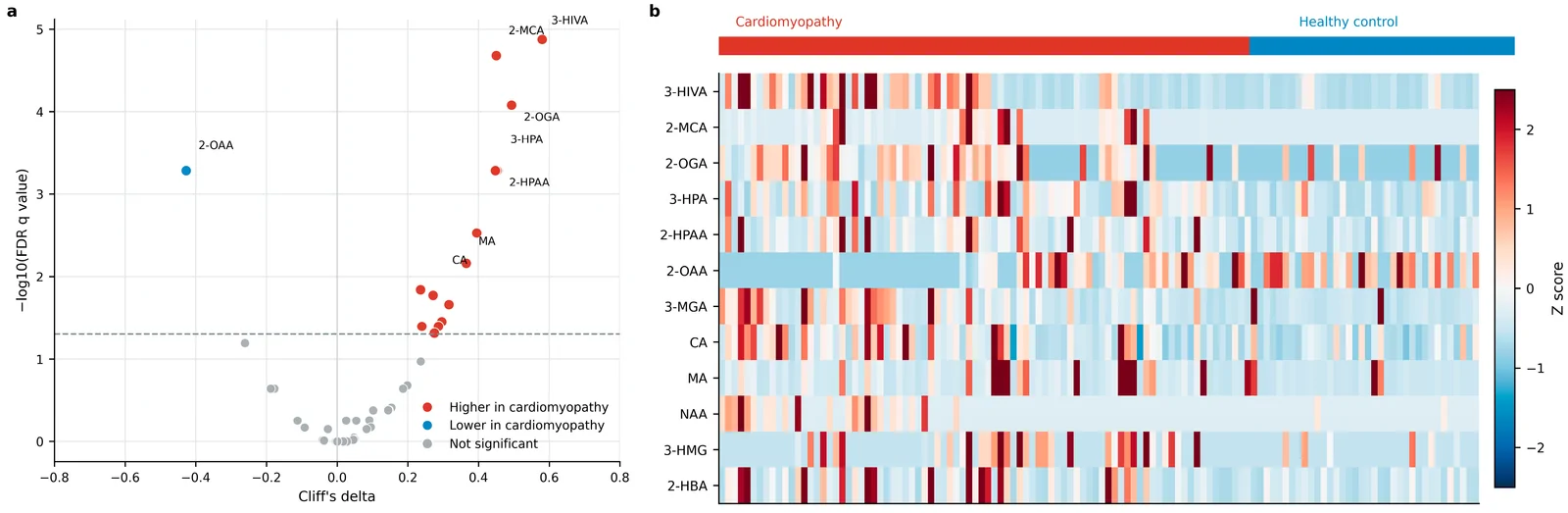
Differential organic acid profile in cardiomyopathy and control participants. (**a**) Volcano plot showing the magnitude and direction of between-group differences across measured organic acids. The x-axis represents Cliff’s delta, and the y-axis represents the negative logarithm of the false discovery rate (FDR)-adjusted q value. Metabolites with positive Cliff’s delta values are increased in cardiomyopathy, whereas those with negative Cliff’s delta values are decreased in cardiomyopathy. The dashed horizontal line indicates the significance threshold after FDR correction. (**b**) Heatmap of the top 12 significantly altered metabolites. Rows represent metabolites and columns represent individual samples. Relative abundances are z-score-normalized by metabolite, with warmer colors indicating higher relative abundance and cooler colors indicating lower relative abundance. Samples are grouped according to cardiomyopathy and control status.

Targeted LC-MS/MS profiling showed an altered circulating organic acid pattern in cardiomyopathy. After Benjamini–Hochberg false discovery rate (FDR) correction, 16 metabolites remained significantly different between groups in the primary unadjusted analysis (Table 2; Supplementary Table S1; Figure 1a). Of these, 15 metabolites were increased in cardiomyopathy, whereas 2-oxoadipic acid was the only metabolite that was decreased relative to control participants. Cliff’s delta values ranged from −0.427 to 0.580, indicating small-to-moderate to moderate between-group separation. Because comorbidities, renal function, glycemic variables, and medication use differed between groups, these findings should not be interpreted as cardiomyopathy-specific effects independent of clinical confounding.

The strongest difference was observed for 3-hydroxyisovaleric acid (median 0.126 [Q1–Q3 0.038–0.424] vs. 0.023 [0.011–0.047]; Cliff’s delta = 0.580; FDR q = 1.33 × 10^−5^), followed by 2-oxoglutaric acid (0.141 [0.017–0.229] vs. 0.000 [0.000–0.000]; Cliff’s delta = 0.493; q = 8.37 × 10^−5^) and 2-methylcitric acid (0.000 [0.000–0.010] vs. 0.000 [0.000–0.000]; Cliff’s delta = 0.450; q = 2.09 × 10^−5^). Additional metabolites that were significantly higher in cardiomyopathy included 3-hydroxypentanoic acid, 2-hydroxyphenylacetic acid, 3-methylglutaconic acid, citric acid, malic acid, N-acetylaspartic acid, 3-hydroxy-3-methylglutaric acid, 2-hydroxybutyric acid, 3-hydroxybutyric acid, 3-hydroxyisobutyric acid, fumaric acid, and suberic acid. In contrast, 2-oxoadipic acid was lower in the cardiomyopathy group (0.000 [0.000–0.099] vs. 0.148 [0.023–0.216]; Cliff’s delta = −0.427; q = 5.20 × 10^−4^).

### 3.3. Global Metabolite Pattern

The effect size-significance plot demonstrated that the most significant metabolites were shifted toward positive Cliff’s delta values, consistent with higher relative abundance in cardiomyopathy (Figure 1a). The accompanying heatmap of the top 12 FDR-significant metabolites further showed a group-related distribution pattern across individual samples (Figure 1b). Row-wise standardized values were generally higher in the cardiomyopathy group for most metabolites, whereas 2-oxoadipic acid displayed the opposite trend. Taken together, these visualizations support a circulating metabolic distinction between cardiomyopathy and control participants, while remaining subject to the baseline clinical differences between groups.

### 3.4. Representative Metabolite Distributions

**Figure 2:**
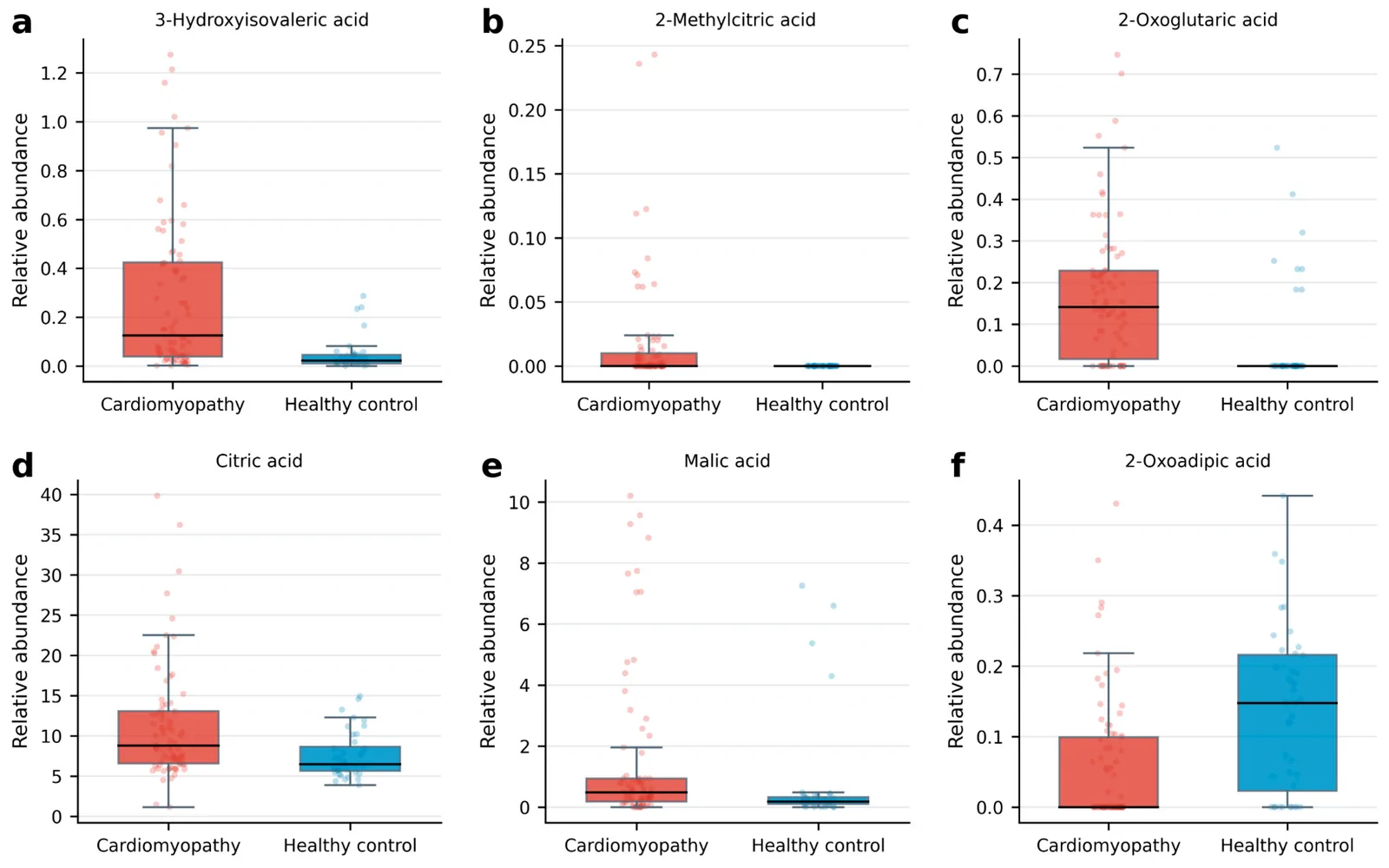
Boxplots of selected significantly altered metabolites. Boxplots showing the distribution of selected significantly altered metabolites in cardiomyopathy and control participants: (**a**) 3-Hydroxyisovaleric acid, (**b**) 2-Methylcitric acid, (**c**) 2-Oxoglutaric acid, (**d**) Citric acid, (**e**) Malic acid, and (**f**) 2-Oxoadipic acid. Center lines indicate the median, boxes indicate the interquartile range, and whiskers indicate the range within 1.5× the interquartile range. Individual dots represent single-sample values.

To illustrate the most representative between-group differences, box-and-whisker plots were generated for six selected metabolites (Figure 2). 3-Hydroxyisovaleric acid, 2-methylcitric acid, 2-oxoglutaric acid, citric acid, and malic acid were all higher in cardiomyopathy, whereas 2-oxoadipic acid was lower. Among these representative metabolites, citric acid was increased from 6.472 (5.639–8.648) in control participants to 8.779 (6.561–13.065) in cardiomyopathy (Cliff’s delta = 0.395; FDR q = 0.003), while malic acid increased from 0.174 (0.100–0.327) to 0.481 (0.174–0.935) (Cliff’s delta = 0.365; FDR q = 0.007). These distributions indicate that the observed differences were driven by shifts in both central tendency and upper-range values.

### 3.5. Exploratory Discriminatory Performance of Selected Metabolites

To further assess exploratory discriminatory performance, receiver operating characteristic (ROC) curve analysis was performed for 3-hydroxyisovaleric acid, 2-oxoglutaric acid, 2-methylcitric acid, and 2-oxoadipic acid. The single-metabolite AUCs were 0.790 for 3-hydroxyisovaleric acid, 0.747 for 2-oxoglutaric acid, 0.725 for 2-methylcitric acid, and 0.714 for 2-oxoadipic acid. The exploratory four-metabolite panel yielded a 5-fold cross-validated AUC of 0.810. These findings support the discriminatory signal of the selected metabolites; however, because the ROC models did not include BMI, renal function, glycemic status, comorbidities, or medication use as covariates, the observed discrimination should be interpreted as exploratory and supportive rather than as formal diagnostic validation or evidence of independence from baseline clinical differences. A targeted summary of the four selected metabolites, including rank-based comparisons, effect sizes, detection rates, and single-metabolite AUCs, is provided in Supplementary Table S3.

### 3.6. Detection Rates and Sensitivity Analyses for Below-LOD Values

Because several significant metabolites had median values of 0.000 in one or both groups, detection rates were examined to distinguish distributional concentration differences from differences in detectability. Among the metabolites with frequent below-LOD values, detection was higher in cardiomyopathy than in control participants for 2-methylcitric acid (45.0% vs. 0.0%), 2-oxoglutaric acid (75.0% vs. 20.0%), N-acetylaspartic acid (27.5% vs. 5.0%), 3-hydroxy-3-methylglutaric acid (40.0% vs. 15.0%), and fumaric acid (42.5% vs. 15.0%). Thus, for these metabolites, the Mann–Whitney results partly reflect a higher proportion of detectable measurements in the cardiomyopathy group. In contrast, 3-hydroxyisovaleric acid, citric acid, malic acid, and suberic acid were detected in nearly all or all samples, supporting interpretation as broader distributional shifts. For highly censored metabolites, detection-frequency results were considered more interpretable than concentration-based comparisons. Detailed detection-rate results are provided in Supplementary Table S2.

### 3.7. Overall Summary of Metabolite Alterations

Overall, cardiomyopathy was characterized by a predominantly upward shift in circulating organic acids, particularly in metabolites linked to intermediary and mitochondrial metabolism, together with a reduction in 2-oxoadipic acid. The findings support an altered circulating organic acid pattern associated with the cardiomyopathy phenotype, but the observed signature may also reflect comorbid renal, glycemic, and treatment-related influences.

A case–control study of patients with non-ischaemic cardiomyopathy and control participants was performed using targeted LC-MS/MS-based organic acid profiling. Differential metabolite patterns were identified, involving pathways related to mitochondrial metabolism, branched-chain amino acids, ketone bodies, and the TCA cycle. ROC analysis indicated moderate exploratory discriminatory performance of selected metabolites, but the findings require validation in clinically adjusted cohorts.

**Figure 3:**
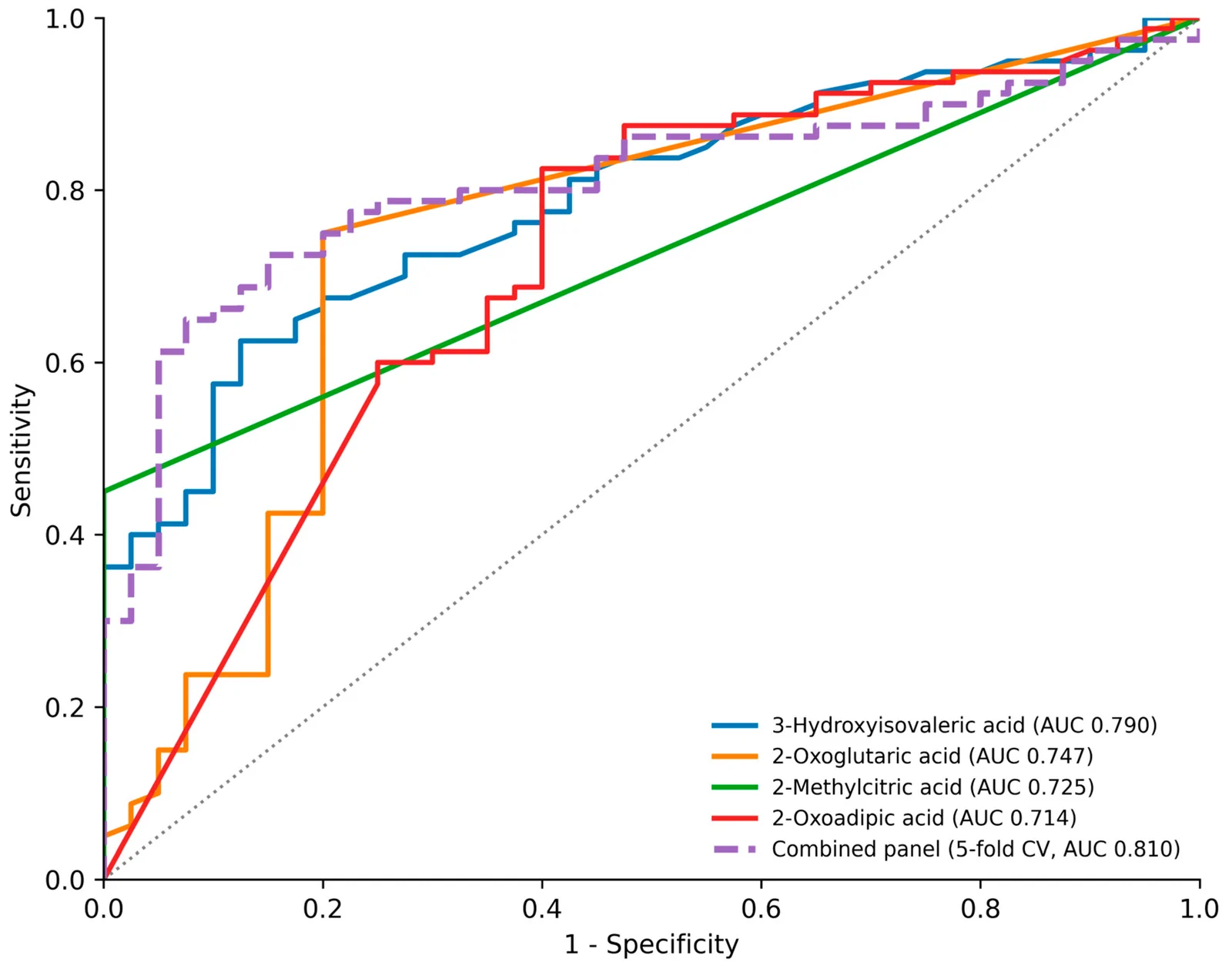
Receiver operating characteristic (ROC) curves for selected discriminatory metabolites in cardiomyopathy. ROC curves were generated for 3-hydroxyisovaleric acid, 2-oxoglutaric acid, 2-methylcitric acid, and 2-oxoadipic acid, together with an exploratory combined four-metabolite panel derived using 5-fold cross-validated logistic regression. The combined panel showed the highest discriminatory performance, followed by 3-hydroxyisovaleric acid. Because metabolite selection was based on the present dataset and several metabolites had values below the limit of detection, these analyses are presented as supportive discrimination analyses and should not be interpreted as formal diagnostic validation.

To further evaluate the discriminatory performance of the selected metabolites, exploratory receiver operating characteristic (ROC) curve analyses were performed for each metabolite and for the combined four-metabolite model. As shown in Figure 3, the combined model demonstrated the highest discriminatory performance (AUC = 0.810), followed by 3-hydroxyisovaleric acid (AUC = 0.790), 2-oxoglutaric acid (AUC = 0.747), 2-methylcitric acid (AUC = 0.725), and 2-oxoadipic acid (AUC = 0.714). These findings should be interpreted as exploratory because the models were not evaluated in an independent validation cohort.

## 4. Discussion

In this case–control LC-MS/MS study, we identified an altered circulating organic acid profile in cardiomyopathy. Sixteen metabolites remained significant after FDR correction, with fifteen showing higher relative abundance and only 2-oxoadipic acid showing lower relative abundance in the cardiomyopathy group. Among the strongest discriminatory metabolites were 3-hydroxyisovaleric acid, 2-methylcitric acid, and 2-oxoglutaric acid. However, because cardiometabolic comorbidities, renal indices, and heart failure therapies differed substantially between groups, these findings should be interpreted as unadjusted phenotype-associated metabolic signals rather than cardiomyopathy-specific causal effects.

A prominent feature of the observed profile was the increase in 3-hydroxyisovaleric acid, accompanied by elevations in several metabolites linked to intermediary substrate turnover. Although our panel was focused on organic acids rather than full amino acid flux analysis, this pattern is compatible with altered branched-chain amino acid-related catabolism. This interpretation is biologically plausible, as impaired branched-chain amino acid metabolism has been increasingly linked to adverse cardiac remodeling, energetic stress, and heart failure progression [15,16,17]. The present findings therefore support the concept that disturbed substrate handling may be a component of the cardiomyopathy-associated metabolic phenotype, while acknowledging that diabetes, insulin resistance, renal function, and medications can influence the same pathways.

We also observed increases in tricarboxylic acid cycle-related metabolites, including 2-oxoglutaric acid, citric acid, malic acid, and fumaric acid. Rather than indicating enhanced oxidative efficiency, this pattern may reflect altered mitochondrial flux, impaired downstream utilization, compensatory accumulation of intermediary metabolites, or systemic effects related to comorbidities and treatment. Prior metabolomics studies have similarly linked citric acid cycle intermediates to cardiovascular risk and heart failure-associated metabolic derangement [18,19]. In this context, our results support the presence of disrupted central carbon metabolism in the cardiomyopathy clinical phenotype, but they do not establish myocardial specificity.

The isolated reduction in 2-oxoadipic acid is also noteworthy. As 2-oxoadipate is connected to lysine and tryptophan degradation and functionally linked to mitochondrial dehydrogenase systems, a decrease in this metabolite may reflect selective perturbation of oxodicarboxylate handling rather than a uniform increase in all mitochondrial intermediates [20,21]. This is of interest because it contrasts with the broader upward shift observed across most other significant organic acids and suggests pathway-specific metabolic dysregulation.

From a clinical and translational perspective, our study extends previous heart failure and cardiomyopathy metabolomics work by applying a focused organic acid-centered approach. Earlier studies have generally used broader metabolite panels, whereas the present design provides a narrower but more biochemically interpretable view of metabolic remodeling [19,22]. This may be valuable when the aim is mechanistic hypothesis generation. Nevertheless, the cardiomyopathy group had higher rates of hypertension, diabetes, dyslipidemia, chronic kidney disease, coronary artery disease, and heart failure medication use. These factors can influence organic acid concentrations through renal clearance, glycemic state, substrate availability, and drug-related metabolic effects. Accordingly, the present findings should be considered descriptive and hypothesis-generating until confirmed in cohorts with individual-level covariate adjustment, propensity methods, or matched controls.

Several limitations should be acknowledged. First, the case–control design does not permit causal inference. Second, changes in circulating metabolites cannot be assumed to directly reflect myocardial intracellular metabolism. Third, the primary metabolite comparisons were unadjusted for important baseline differences, including renal function, diabetes, dyslipidemia, coronary artery disease, and medication use; residual confounding is therefore likely. Fourth, several metabolites had frequent below-LOD values, meaning that some significant Mann–Whitney results partly reflect differences in detectability rather than concentration alone. Fifth, exact fasting duration, time of day, storage duration, batch randomization, detailed echocardiographic severity measures, and cardiomyopathy subtype descriptors were not uniformly available in the analysis dataset. Sixth, although the selected metabolites showed consistent discriminatory performance, the ROC analyses were exploratory and should not be interpreted as formal diagnostic validation or as evidence that the metabolite signal is independent of baseline clinical differences such as BMI, renal function, glycemic status, comorbidities, or medication use. The study has strengths, including targeted LC-MS/MS quantification, FDR correction, effect size-based interpretation, and transparent detection-rate sensitivity analyses. In addition, the absence of an external validation cohort limits the generalizability of the findings. To the best of our knowledge, this is among the first targeted LC-MS/MS-based studies specifically focusing on circulating organic acid profiles in non-ischemic cardiomyopathy.

## 5. Conclusions

Cardiomyopathy was associated with an altered circulating organic acid pattern characterized predominantly by elevations in intermediary metabolites and a reduction in 2-oxoadipic acid. This circulating pattern is compatible with alterations in mitochondrial and substrate-related metabolic pathways; however, it should be interpreted in the context of comorbidity, renal function, glycemic status, and treatment-related confounding. Larger prospective studies with individual-level covariate adjustment, detailed echocardiographic phenotyping, and external validation are needed to determine whether this metabolic profile has incremental value for phenotypic stratification, disease monitoring, or treatment–response assessment.

## Figures and Tables

**Table 1:** Baseline demographic, clinical, and laboratory characteristics of the study population.

Variable	Cardiomyopathy (*n* = 80)	Control Participants (*n* = 80)	*p* Value
Demographic characteristics			
Age, years	58.6 ± 12.4	56.9 ± 11.8	0.387
Female sex, *n* (%)	28 (35.0)	30 (37.5)	0.741
Body mass index, kg/m^2^	27.8 ± 4.6	26.9 ± 3.9	0.184
Current smoking, *n* (%)	18 (22.5)	12 (15.0)	0.228
Systolic blood pressure, mmHg	122 ± 16	119 ± 13	0.196
Diastolic blood pressure, mmHg	76 ± 10	74 ± 8	0.161
Heart rate, beats/min	78 ± 12	72 ± 9	0.001
Clinical history and comorbidities			
Hypertension, *n* (%)	46 (57.5)	18 (22.5)	<0.001
Diabetes mellitus, *n* (%)	24 (30.0)	8 (10.0)	0.002
Dyslipidemia, *n* (%)	34 (42.5)	14 (17.5)	0.001
Coronary artery disease, *n* (%)	20 (25.0)	2 (2.5)	<0.001
Atrial fibrillation, *n* (%)	12 (15.0)	1 (1.3)	0.002
Chronic kidney disease, *n* (%)	10 (12.5)	2 (2.5)	0.017
Cardiomyopathy etiology			
Non-ischemic cardiomyopathy (NICM), *n* (%)	80 (100)	—	—
Ischemic cardiomyopathy excluded	Yes	—	—
Routine laboratory variables			
Hemoglobin, g/dL	13.4 ± 1.7	14.1 ± 1.3	0.005
White blood cell count, ×10^9^/L	7.6 ± 2.1	6.8 ± 1.7	0.011
Creatinine, mg/dL	0.98 (0.82–1.22)	0.84 (0.73–0.95)	0.003
Estimated GFR, mL/min/1.73 m^2^	78.4 ± 19.6	92.7 ± 14.8	<0.001
Fasting glucose, mg/dL	104 ± 24	92 ± 12	<0.001
AST, U/L	24 (19–31)	21 (18–26)	0.041
ALT, U/L	23 (17–31)	20 (16–25)	0.079
Total cholesterol, mg/dL	181 ± 42	192 ± 36	0.071
LDL-cholesterol, mg/dL	109 ± 31	116 ± 28	0.108
HDL-cholesterol, mg/dL	41 ± 11	49 ± 12	<0.001
Triglycerides, mg/dL	156 (112–208)	118 (89–154)	0.002
Medication use			
ACE inhibitor or ARB, *n* (%)	48 (60.0)	6 (7.5)	<0.001
ARNI, *n* (%)	18 (22.5)	0 (0.0)	<0.001
Beta-blocker, *n* (%)	58 (72.5)	5 (6.3)	<0.001
Mineralocorticoid receptor antagonist, *n* (%)	28 (35.0)	0 (0.0)	<0.001
SGLT2 inhibitor, *n* (%)	16 (20.0)	1 (1.3)	<0.001
Loop diuretic, *n* (%)	36 (45.0)	0 (0.0)	<0.001
Statin, *n* (%)	30 (37.5)	10 (12.5)	<0.001
Antiplatelet therapy, *n* (%)	24 (30.0)	4 (5.0)	<0.001
Oral anticoagulant, *n* (%)	14 (17.5)	1 (1.3)	<0.001

Data are presented as mean ± standard deviation, median (interquartile range), or n (%), as appropriate. Between-group comparisons were performed using the independent-samples *t* test or Mann–Whitney *U* test for continuous variables and the chi-square test or Fisher’s exact test for categorical variables, as appropriate. ACE, angiotensin-converting enzyme; ALT, alanine aminotransferase; ARB, angiotensin receptor blocker; ARNI, angiotensin receptor–neprilysin inhibitor; AST, aspartate aminotransferase; GFR, glomerular filtration rate; HDL, high-density lipoprotein; LDL, low-density lipoprotein; SGLT2, sodium-glucose cotransporter 2.

**Table 2:** Significantly altered organic acids in cardiomyopathy versus control participants.

Metabolite	Cardiomyopathy, Median (Q1–Q3)	Control, Median (Q1–Q3)	Cliff’s Delta	FDR q-Value
3-Hydroxyisovaleric acid	0.126 (0.038–0.424)	0.023 (0.011–0.047)	0.580	1.33 × 10^−5^
2-Methylcitric acid	0.000 (0.000–0.010)	0.000 (0.000–0.000)	0.450	2.09 × 10^−5^
2-Oxoglutaric acid	0.141 (0.017–0.229)	0.000 (0.000–0.000)	0.493	8.37 × 10^−5^
3-Hydroxypentanoic acid	0.121 (0.048–0.240)	0.039 (0.019–0.101)	0.455	5.20 × 10^−4^
2-Hydroxyphenylacetic acid	0.005 (0.002–0.007)	0.002 (0.001–0.004)	0.452	5.20 × 10^−4^
2-Oxoadipic acid	0.000 (0.000–0.099)	0.148 (0.023–0.216)	−0.427	5.20 × 10^−4^
3-Methylglutaconic acid	0.059 (0.020–0.163)	0.020 (0.014–0.029)	0.448	5.20 × 10^−4^
Citric acid	8.779 (6.561–13.065)	6.472 (5.639–8.648)	0.395	0.003
Malic acid	0.481 (0.174–0.935)	0.174 (0.100–0.327)	0.365	0.007
N-acetylaspartic acid	0.000 (0.000–0.004)	0.000 (0.000–0.000)	0.236	0.015
3-Hydroxy-3-methylglutaric acid	0.000 (0.000–0.005)	0.000 (0.000–0.000)	0.271	0.017
2-Hydroxybutyric acid	1.563 (0.687–4.140)	0.899 (0.397–1.538)	0.316	0.022
3-Hydroxybutyric acid	1.178 (0.449–5.096)	0.676 (0.330–1.420)	0.296	0.035
3-Hydroxyisobutyric acid	0.289 (0.150–0.524)	0.193 (0.085–0.293)	0.287	0.040
Fumaric acid	0.000 (0.000–0.074)	0.000 (0.000–0.000)	0.239	0.040
Suberic acid	0.929 (0.596–1.096)	0.734 (0.539–0.969)	0.275	0.049

Note: Data are presented as median (interquartile range [Q1–Q3]). Between-group comparisons were performed using the two-sided Mann–Whitney *U* test. To account for multiple comparisons, *p*-values were adjusted using the Benjamini–Hochberg false discovery rate method, and the corresponding q-values are reported. Cliff’s delta is provided as a measure of effect size; positive values indicate higher concentrations in the cardiomyopathy group, whereas negative values indicate lower concentrations in the cardiomyopathy group. q < 0.05 was considered statistically significant. Concentrations are expressed in relative abundance units derived from the targeted LC–MS/MS assay.

## Data Availability

The data supporting the findings of this study are available from the corresponding author upon reasonable request. The data are not publicly available due to privacy and ethical restrictions.

## Supplementary Materials

The following supporting information can be downloaded at https://www.mdpi.com/article/10.3390/jcdd13080350/s1, Table S1: FDR-significant circulating organic acids in cardiomyopathy and control participants; Table S2: Detection rates for FDR-significant metabolites; Table S3: Targeted sensitivity analysis of four selected metabolites.

## Abbreviations

The following abbreviations are used in this manuscript:

ACE	Angiotensin-Converting Enzyme
ALT	Alanine Aminotransferase
ARB	Angiotensin Receptor Blocker
ARNI	Angiotensin Receptor–Neprilysin Inhibitor
AST	Aspartate Aminotransferase
AUC	Area Under the Curve
BMI	Body Mass Index
CAD	Coronary Artery Disease
FDR	False Discovery Rate
GFR	Glomerular Filtration Rate
HDL	High-Density Lipoprotein
HPLC	High-Performance Liquid Chromatography
LC–MS/MS	Liquid Chromatography–Tandem Mass Spectrometry
LDL	Low-Density Lipoprotein
LOD	Limit of Detection
MRM	Multiple Reaction Monitoring
NICM	Non-Ischemic Cardiomyopathy
ROC	Receiver Operating Characteristic
SGLT2	Sodium–Glucose Cotransporter 2
STROBE	Strengthening the Reporting of Observational Studies in Epidemiology
