# Supplementary material for: Circulating Organic Acid Profiles in Non-Ischaemic Cardiomyopathy: A Case–Control Study
Source: J Cardiovasc Dev Dis. 2026 Jul 27;13(8):350. doi: 10.3390/jcdd13080350 (PMC13513701; doi:10.3390/jcdd13080350)
Supplement: Supplementary file 1 [file jcdd-13-00350-s001.zip › jcdd-4312870-supplementary.pdf]

Supplementary Table S1. FDR-significant circulating organic acids in cardiomyopathy and control participants.

Rank	Metabolite	Direction in cardiomyopathy	Cardiomyopathy, median (Q1-Q3)	Control, median (Q1-Q3)	Cliff's delta	FDR q-value
**1**	3-Hydroxyisovaleric acid	Higher	0.126 (0.038-0.424)	0.023 (0.011-0.047)	0.580	1.33e-05
**2**	2-Methylcitric acid	Higher	0.000 (0.000-0.010)	0.000 (0.000-0.000)	0.450	2.09e-05
**3**	2-Oxoglutaric acid	Higher	0.141 (0.017-0.229)	0.000 (0.000-0.000)	0.493	8.37e-05
**4**	3-Hydroxypentanoic acid	Higher	0.121 (0.048-0.240)	0.039 (0.019-0.101)	0.455	5.20e-04
**5**	2-Hydroxyphenylacetic acid	Higher	0.005 (0.002-0.007)	0.002 (0.001-0.004)	0.452	5.20e-04
**6**	2-Oxoadipic acid	Lower	0.000 (0.000-0.099)	0.148 (0.023-0.216)	-0.427	5.20e-04
**7**	3-Methylglutaconic acid	Higher	0.059 (0.020-0.163)	0.020 (0.014-0.029)	0.448	5.20e-04
**8**	Citric acid	Higher	8.779 (6.561-13.065)	6.472 (5.639-8.648)	0.395	0.003
**9**	Malic acid	Higher	0.481 (0.174-0.935)	0.174 (0.100-0.327)	0.365	0.007
**10**	N-acetylaspartic acid	Higher	0.000 (0.000-0.004)	0.000 (0.000-0.000)	0.236	0.015
**11**	3-Hydroxy-3-methylglutaric acid	Higher	0.000 (0.000-0.005)	0.000 (0.000-0.000)	0.271	0.017
**12**	2-Hydroxybutyric acid	Higher	1.563 (0.687-4.140)	0.899 (0.397-1.538)	0.316	0.022
**13**	3-Hydroxybutyric acid	Higher	1.178 (0.449-5.096)	0.676 (0.330-1.420)	0.296	0.035
**14**	3-Hydroxyisobutyric acid	Higher	0.289 (0.150-0.524)	0.193 (0.085-0.293)	0.287	0.040
**15**	Fumaric acid	Higher	0.000 (0.000-0.074)	0.000 (0.000-0.000)	0.239	0.040
**16**	Suberic acid	Higher	0.929 (0.596-1.096)	0.734 (0.539-0.969)	0.275	0.049

Metabolites are ranked by FDR q-value (ascending).

Data are shown as median (Q1-Q3). Group comparisons were performed using the two-sided Mann-Whitney U test. q-values were adjusted with the Benjamini-Hochberg false discovery rate method. Positive Cliff's delta values indicate higher concentrations in cardiomyopathy, whereas negative values indicate lower concentrations in cardiomyopathy. Only metabolites with q < 0.05 are shown.

Supplementary Table S2. Detection rates for FDR-significant metabolites.

Metabolite	Cardiomyopathy detected, n/N (%)	Control participants detected, n/N (%)	FDR q for detection
**3-Hydroxyisovaleric acid**	80/80 (100.0)	80/80 (100.0)	1.000
**2-Methylcitric acid**	36/80 (45.0)	0/80 (0.0)	5.65e-07
**2-Oxoglutaric acid**	60/80 (75.0)	16/80 (20.0)	5.65e-07
**3-Hydroxypentanoic acid**	80/80 (100.0)	80/80 (100.0)	1.000

**2-Hydroxyphenylacetic acid**	80/80 (100.0)	68/80 (85.0)	0.011
**2-Oxoadipic acid**	34/80 (42.5)	60/80 (75.0)	0.011
**3-Methylglutaconic acid**	72/80 (90.0)	72/80 (90.0)	1.000
**Citric acid**	80/80 (100.0)	80/80 (100.0)	1.000
**Malic acid**	72/80 (90.0)	68/80 (85.0)	1.000
**N-acetylaspartic acid**	22/80 (27.5)	4/80 (5.0)	0.025
**3-Hydroxy-3-methylglutaric acid**	32/80 (40.0)	12/80 (15.0)	0.039
**2-Hydroxybutyric acid**	74/80 (92.5)	72/80 (90.0)	1.000
**3-Hydroxybutyric acid**	74/80 (92.5)	76/80 (95.0)	1.000
**3-Hydroxyisobutyric acid**	68/80 (85.0)	72/80 (90.0)	1.000
**Fumaric acid**	34/80 (42.5)	12/80 (15.0)	0.025
**Suberic acid**	80/80 (100.0)	80/80 (100.0)	1.000

Supplementary Table S3. Targeted sensitivity analysis of four selected metabolites.

**Metabolite**	**Direction**	**CM median (Q1-Q3)**	**Control median (Q1-Q3)**	**Cliff's delta**	**Mann-Whitney U p**	**Full-panel FDR q**	**CM detected, n/N (%)**	**Control detected, n/N (%)**	**Detection Fisher p**	**Detection FDR q**	**AUC**
**3-Hydroxyisovaleric acid**	Higher in CM	0.126 (0.038-0.424)	0.023 (0.011-0.047)	0.580	2.46e-10	1.33e-05	80/80 (100.0)	80/80 (100.0)	1.000	1.000	0.790
**2-Oxoglutaric acid**	Higher in CM	0.141 (0.017-0.229)	0.000 (0.000-0.000)	0.493	5.85e-09	8.37e-05	60/80 (75.0)	16/80 (20.0)	2.75e-12	5.65e-07	0.747
**2-Methylcitric acid**	Higher in CM	0.000 (0.000-0.010)	0.000 (0.000-0.000)	0.450	1.83e-11	2.09e-05	36/80 (45.0)	0/80 (0.0)	1.72e-13	5.65e-07	0.725
**2-Oxoadipic acid**	Lower in CM	0.000 (0.000-0.099)	0.148 (0.023-0.216)	-0.427	1.32e-06	5.20e-04	34/80 (42.5)	60/80 (75.0)	5.08e-05	0.011	0.714

Supplementary Table S3. Targeted sensitivity analysis of four selected metabolites. Between-group differences were assessed using the two-sided Mann-Whitney U test; the full-panel FDR q values are the corresponding Benjamini-Hochberg-adjusted values reported in Table 2 and were not recalculated across only the four selected rows. Effect sizes are reported as Cliff's delta. Detection rates were defined as the proportion of samples with measurable values greater than zero; detection FDR q values correspond to the full detection-rate analysis reported in Supplementary Table 2. AUC values were derived from exploratory single-metabolite ROC analyses. These analyses are intended as supportive sensitivity analyses and should not be interpreted as formal diagnostic validation.
